# Supplementary material for: HAMMER: Hairpin-based APOBEC3A-mediated mRNA editing reporter
Source: bioRxiv. 2026 Feb 15:2025.12.22.695965. Preprint. [Version 2] doi: 10.64898/2025.12.22.695965 (PMC12918910; doi:10.64898/2025.12.22.695965)
Supplement: Supplement 1 [file media-1.pdf]

Supplementary Materials for:

HAMMER: Hairpin-based APOBEC3A-mediated mRNA editing reporter

Yanjun Chen[1], Christopher D. Mullally[1], Bojana Stefanovska[1,2], Reuben S. Harris[1,2,*]

[1] Department of Biochemistry and Structural Biology, University of Texas San Antonio, San Antonio, Texas, USA, 78229

[2] Howard Hughes Medical Institute, University of Texas San Antonio, San Antonio, Texas, USA, 78229

* Correspondence to: rsh@uthscsa.edu

Supplementary Tables S1-S3

Supplementary Figures S1-S4

Table S1. gBlocks.

Name	Sequence 5' to 3'
DDOST hairpin1 linker	AAGCTTCGTGGCAGACACCGAGAACCTGCTGAAGGCCCCATCCATCGATGGGAAATAAAGTGTAAAGAACATACTCTAGAGGGCCCA
DDOST hairpin2 gBlock	GAGCGCGTGCTGAAGAACGAGCAGAAGCTTCGTGGCAGACACCGAGAACCTGCTGAAGGCACCCATCATCGATGGGAAATAAAGTGTAAAGAACATACTCTAGAGGGCCCAGAAGACGCCAAAAACATA
DDOST hairpin3 gBlock	GAGCGCGTGCTGAAGAACGAGCAGAAGCTTCGTGGCAGACACCGAGAACCTGCTGAAGGCACCCATCTTCGATGGGAAATAAAGTGTAAAGAACATACTCTAGAGGGCCCAGAAGACGCCAAAAACATA
DDOST hairpin4 gBlock	GAGCGCGTGCTGAAGAACGAGCAGAAGCTTCGTGGCAGACACCGAGAACCTGGTTTAGGCCCCATCCATCGATGGGGCCTAAAGTGTAAAGAACATACTCTAGAGGGCCCAGAAGACGCCAAAAACATA
DDOST hairpin5 gBlock	GAGCGCGTGCTGAAGAACGAGCAGAAGCTTCGTGGCAGACACCGAGAACCTGGATTTAGGCCCCATCATCGATGGGGCCTAAAGTGTAAAGAACATACTCTAGAGGGCCCAGAAGACGCCAAAAACATA
DDOST hairpin6 gBlock	GAGCGCGTGCTGAAGAACGAGCAGAAGCTTCGTGGCAGACACCGAGAACCTGGATTTAGGCCCCATCTTCGATGGGGCCTAAAGTGTAAAGAACATACTCTAGAGGGCCCAGAAGACGCCAAAAACATA
DDOST hairpin7 gBlock	GAGCGCGTGCTGAAGAACGAGCAGAAGCTTCGTGGCAGACACCGAGAACTTGGTTTAGGCCCCATCCATCGATGGGGCCTAAACCAAAAAGAACATACTCTAGAGGGCCCAGAAGACGCCAAAAACATA
DDOST hairpin8 gBlock	GAGCGCGTGCTGAAGAACGAGCAGAAGCTTCGTGGCAGACACCGAGAAACTTGGTTTAGGCCCCATCATCGATGGGGCCTAAACCAAAAAGAACATACTCTAGAGGGCCCAGAAGACGCCAAAAACATA
DDOST hairpin9 gBlock	GAGCGCGTGCTGAAGAACGAGCAGAAGCTTCGTGGCAGACACCGAGAAACTTGGTTTAGGCCCCATCTTCGATGGGGCCTAAACCAAAAAGAACATACTCTAGAGGGCCCAGAAGACGCCAAAAACATA
CYFIP1 hairpin gBlock	GAGCGCGTGCTGAAGAACGAGCAGAAGCTTCGTGGCAGACACCGAGAACCTGCTGAAGGAAATTTCCATCGAAAGAGAATAAAGTGTAAAGAACATACTCTAGAGGGCCCAGAAGACGCCAAAAACATA
SDHB hairpin gBlock	GAGCGCGTGCTGAAGAACGAGCAGAAGCTTCGTGGCAGACACCGAGAACCTGCTGAAGGCACCATCTATCGATGGGAAATAAAGTGTAAAGAACATACTCTAGAGGGCCCAGAAGACGCCAAAAACATA
NUP93 hairpin gBlock	GAGCGCGTGCTGAAGAACGAGCAGAAGCTTCGTGGCAGACACCGAGAACCTGCTGATCAGCAAGCTCATCAGCTTGCTGTAAAGTGTAAAGAACATACTCTAGAGGGCCCAGAAGACGCCAAAAACATA
MD21D2 hairpin gBlock	GAGCGCGTGCTGAAGAACGAGCAGAAGCTTCGTGGCAGACACCGAGAACCTGCTGAATTGCAGGCCTATCAGGCCTGCATAAAGTGTAAAGAACATACTCTAGAGGGCCCAGAAGACGCCAAAAACATA
FAM83G hairpin gBlock	GAGCGCGTGCTGAAGAACGAGCAGAAGCTTCGTGGCAGACACCGAGAACCTGCTGAATCGGGCCCCTCTCAGGGGCCCGTAAAGTGTAAAGAACATACTCTAGAGGGCCCAGAAGACGCCAAAAACATA

Table S2. Oligonucleotide sequences.

Oligo name	Purpose	Sequence 5' to 3'
Linear1-F	Site-directed mutagenesis for making *DDOST* Linear1 reporter (forward)	CTGAAGGCTTTTTTCATCGATGGGAAATAAAG TGT
Linear1-R	Site-directed mutagenesis for making *DDOST* Linear1 reporter (reverse)	CATCGATGAAAAAAGCCTTCAGCAGGTTCTCG
Stop1-F	Site-directed mutagenesis for making *DDOST* Stop1 reporter (forward)	CCCATCCATTGATGGGAAATAAAGTGT
Stop1-R	Site-directed mutagenesis for making *DDOST* Stop1 reporter (reverse)	CATCAATGGATGGGGCCTTCAGCA
Cas9n-BE4max-F	Site-directed mutagenesis for removal of rAPOBEC1 from BE4max (forward)	GGAAAGTCGACAAGAAGTACAGCATCGGC
Cas9n-BE4max-R	Site-directed mutagenesis for removal of rAPOBEC1 from BE4max (reverse)	TCTTGTCGACTTTCCGCTTCTTCTTTGG
HAMMER-F	PCR HAMMER reporter linker region (forward)	CACTGCATACGACGATTCTGTG
HAMMER-R	PCR HAMMER reporter linker region (reverse); Reverse transcription of HAMMER mRNA	ATGTTCATCGAGTCCGACCC
HAMMER-seq	Sanger sequencing of HAMMER PCR products	TATTGTCGAGGGAGCTAAGA

Table S3. Viral ribonucleotide reductases.

Virus	Accession	Host Species	New World or Old World	Virus Family
HSV1	YP_009137114	Human	Old World	Alphaherpesvirinae
HCMV	YP_081503.1	Human	Old World	Betaherpesvirinae
EBV	YP_001129452.1	Human	Old World	Gammaherpesvirinae - lymphocryptovirus
KSHV	QLI54727.1	Human	Old World	Gammaherpesvirinae - rhadinovirus
CalHV3	NP_733909.1	Marmoset	New World	Gammaherpesvirinae - lymphocryptovirus
SgHV1	UNP64460.1	Marmoset	New World	Gammaherpesvirinae - rhadinovirus
AtHV3	NP_048032.1	Atelinae	New World	Gammaherpesvirinae - rhadinovirus
SaHV2	NP_040263.1	Squirrel Monkey	New World	Gammaherpesvirinae - rhadinovirus
McHV13	YP_010801332.1	Macaque	Old World	Gammaherpesvirinae - lymphocryptovirus
McHV10	YP_010084648.1	Macaque	Old World	Gammaherpesvirinae - lymphocryptovirus
McHV4	YP_067953.1	Macaque	Old World	Gammaherpesvirinae - lymphocryptovirus
McHv5	NP_570809.1	Macaque	Old World	Gammaherpesvirinae - rhadinovirus
McHV8	YP_010084428.1	Macaque	Old World	Gammaherpesvirinae - rhadinovirus

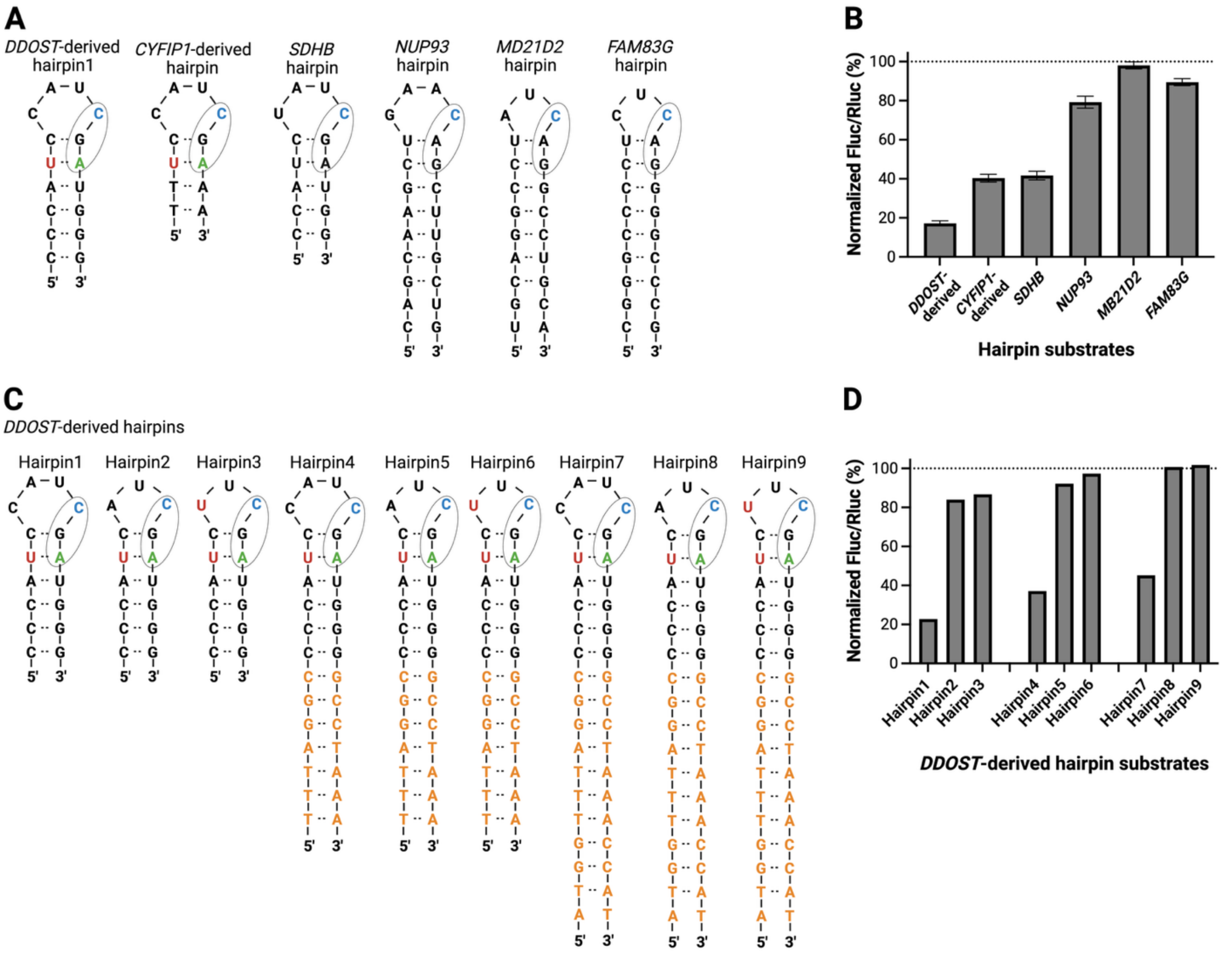

Fig. S1. Additional hairpin substrates tested using HAMMER.

A, Schematics of HAMMER reporter hairpin substrates derived from endogenous A3A-edited transcripts and engineered with variable loop residues and stem lengths. Target cytosines are highlighted in blue; bases modified from original sequences are colored in red (U) and green (A); stem bases extending from original sequences are shaded in orange.

B, Normalized firefly-to-renilla luminescence ratios for the indicated HAMMER reporters with different hairpin substrates co-expressed with human A3A in 293T cells [mean +/- SD of 2 biological reactions normalized to A3A-E72A control (dotted line at 100%)].

C, Schematics of DDOST1 hairpin1 and derivative hairpin substrates tested in the system described here. Target cytosines are highlighted in blue; bases modified from original sequences are colored in red (U) and green (A); stem bases extending from original sequences are shaded in orange.

D, Normalized firefly-to-renilla luminescence ratios for the indicated reporters with different DDOST-derived hairpin substrates co-expressed with human A3A in 293T cells [n=1 experiment normalized to A3A-E72A control (dotted line at 100%)].

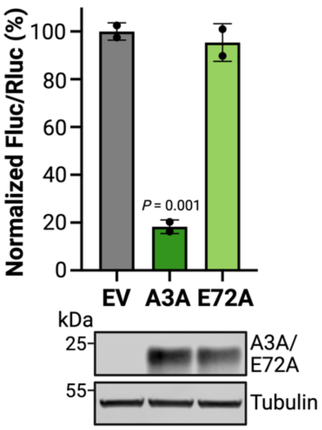

Fig. S2. HAMMER reporter functionality in HeLa.

Normalized firefly-to-renilla luminescence ratios of HeLa cells co-transfected with Hairpin1 reporter and either EV, A3A, or the catalytic mutant A3A-E72A (mean ± SD of 2 biological replicates). Immunoblots below confirm expression of A3A and A3A-E72A, with tubulin as a loading control.

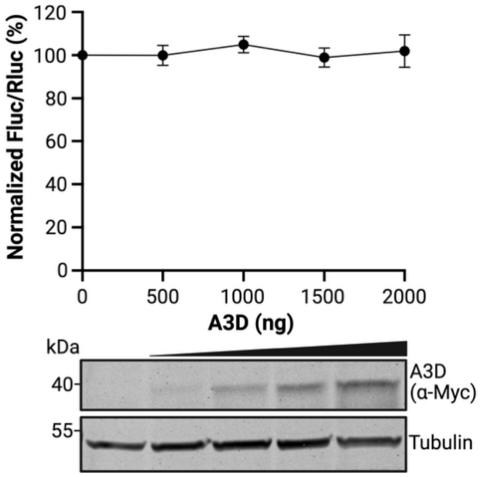

Fig. S3. HAMMER is not a substrate for human A3D in 293T cells.

Normalized firefly-to-renilla luminescence ratios of 293T cells co-transfected with Hairpin1 reporter and increasing amounts of human A3D (mean ± SD of 2 biological replicates). Immunoblots below confirm expression of A3D (anti-Myc) with tubulin as a loading control.

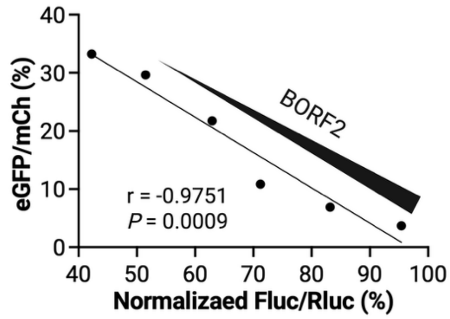

Fig. S4. Relationship between DNA and RNA editing reporter activities.

Correlation between HAMMER luminescence readout (x-axis) and AMBER fluorescence readout (y-axis) across increasing BORF2 expression levels in 293T cells co-expressing a fixed amount of A3A (200 ng) with each reporter. BORF2 was titrated using a two-fold dilution series starting from 400 ng plasmid. Pearson correlation coefficient (r) and corresponding *P*-value are indicated.
